# Comparative Evaluation of U.S. Brand and Generic Intravenous Sodium Ferric Gluconate Complex in Sucrose Injection: Biodistribution after Intravenous Dosing in Rats

**DOI:** 10.3390/nano8010010

**Published:** 2017-12-28

**Authors:** Christopher R. Beekman, Murali K. Matta, Christopher D. Thomas, Adil Mohammad, Sharron Stewart, Lin Xu, Ashok Chockalingam, Katherine Shea, Dajun Sun, Wenlei Jiang, Vikram Patel, Rodney Rouse

**Affiliations:** 1 U. S. Food and Drug Administration, Center for Drug Evaluation and Research, Office of Translational Science, Office of Clinical Pharmacology, Division of Applied Regulatory Science, Silver Spring, MD 20993, USA; christopher.beekman@fda.hhs.gov (C.R.B.); murali.matta@fda.hhs.gov (M.K.M.); thomdc15@wfu.edu (C.D.T.); sharron.stewart@fda.hhs.gov (S.S.); lin.xu@fda.hhs.gov (L.X.); ashok.chockalingam@fda.hhs.gov (A.C.); katherine.shea@fda.hhs.gov (K.S.); Vikram.patel@fda.hhs.gov (V.P.); 2 U. S. Food and Drug Administration, Center for Drug Evaluation and Research, Office of Pharmaceutical Quality, Office of Testing and Research, Division of Product Quality Research, Silver Spring, MD 20993, USA; adil.mohammad@fda.hhs.gov; 3 U. S. Food and Drug Administration, Center for Drug Evaluation and Research, Office of Generic Drugs, Office of Research and Standards, Silver Spring, MD 20993, USA; dajun.sun@fda.hhs.gov (D.S.); wenlei.jiang@fda.hhs.gov (W.J.)

**Keywords:** biodistribution, sodium ferric gluconate complex, rats, bioequivalence, Ferrlecit

## Abstract

Relative biodistribution of FDA-approved innovator and generic sodium ferric gluconate (SFG) drug products was investigated to identify differences in tissue distribution of iron after intravenous dosing to rats. Three equal cohorts of 42 male Sprague-Dawley rats were created with each cohort receiving one of three treatments: (1) the innovator SFG product dosed intravenously at a concentration of 40 mg/kg; (2) the generic SFG product dosed intravenously at a concentration of 40 mg/kg; (3) saline dosed intravenously at equivalent volume to SFG products. Sampling time points were 15 min, 1 h, 8 h, 1 week, two weeks, four weeks, and six weeks post-treatment. Six rats from each group were sacrificed at each time point. Serum, femoral bone marrow, lungs, brain, heart, kidneys, liver, and spleen were harvested and evaluated for total iron concentration by ICP-MS. The ICP-MS analytical method was validated with linearity, range, accuracy, and precision. Results were determined for mean iron concentrations (µg/g) and mean total iron (whole tissue) content (µg/tissue) for each tissue of all groups at each time point. A percent of total distribution to each tissue was calculated for both products. At any given time point, the overall percent iron concentration distribution did not vary between the two SFG drugs by more than 7% in any tissue. Overall, this study demonstrated similar tissue biodistribution for the two SFG products in the examined tissues.

## 1. Introduction

Sodium ferric gluconate (SFG) is a colloidal iron parenteral product indicated for intravenous iron replacement therapy in patients with moderate to severe iron deficiency anemia, a common finding in chronic illness particularly in advanced renal failure. Consequently, SFG is frequently administered concurrently with dialysis and is generally effective in increasing total iron (TI) in the blood and combating iron deficiency anemia [1]. SFG is a complex drug product comprised of ferric oxyhydroxide nanoparticles coated with gluconate suspended in a sucrose solution. Distribution and release of the iron ion from the drug is size and surface dependent, with differences in core size and carbohydrate chemistry determining pharmacologic and bioactivity differences such as clearance rate, iron release rate, maximum tolerated dose, and rate of infusion [2,3,4].

Once administered, SFG nanoparticles are phagocytized by cells of the reticuloendothelial system (RES) where iron ions become part of the intracellular iron pool [5]. The destiny of the iron ions is dependent upon the physiological demand for iron. If iron is in excess, iron ions will be slowly excreted through the urine or bile or stored intracellularly as ferritin or hemosiderin. An immediate need for iron results in mobilization of the iron ions from the cell. To efficiently move iron into and throughout the circulation, it is bound to a transport protein, transferrin [6,7]. Transferrin bound iron (TBI) is carried to the site of iron requirement and released for utilization. Iron overload disorders can arise from genetic iron metabolism dysfunction or excess iron intake or transiently if iron released from an iron replacement therapy exceeds the transferrin binding capacity [1,6]. When transferrin is saturated, non-transferrin bound iron (NTBI) can be found in the blood. NTBI is iron that is non-specifically and loosely bound to blood proteins, sugars or others and that has the potential to participate in oxidation-reduction reactions, create reactive oxygen species and free radicals, and induce oxidative stress [6,8]. Extended oxidative stress can have numerous adverse clinical outcomes including hypotension, diarrhea, chest pain, and functional compromise of multiple organs [6,7]. Thus, one of the safety concerns for SFG drug products is rate of iron release relative to transferrin saturation and the potential for oxidative stress.

In 2013, the U.S. FDA published the draft product-specific guidance for SFG complex injection, recommending in vitro and in vivo studies for demonstrating bioequivalence [9]. Establishing bioequivalence for SFG complex drug products requires qualitative (Q1) and quantitative (Q2) formulation sameness, in vivo bioequivalence pharmacokinetic studies, and thorough characterization of the product to show equivalence in physiochemical properties between reference and test products. In this case, the in vivo bioequivalence pharmacokinetic study measures total iron (TI) in serum and TBI in serum and the bioequivalence is based on 90% confidence interval of maximum value of the difference in concentration between TI and TBI over all time points measured and difference in AUC between TI and TBI. In 2011, the European Medicines Agency (EMA) published a draft reflection paper and further finalized in 2015 recommending non-clinical studies (e.g., characterization of RES uptake, biodistribution (BD) in animal models) in addition to the studies requested by the FDA in order to compare the efficacy and safety profiles between reference and test products [10,11]. To reconcile the different bioequivalence approaches of the two regulatory agencies, a cellular uptake study was completed [12] and this BD study in a rat model evaluated whether a FDA-approved generic SFG complex drug product would have comparable biodistribution in tissues compared to its reference counterpart and would not yield a difference in iron release resulting in a different potential for oxidative stress and therefore a different safety profile.

This manuscript describes a BD study in rats for Ferrlecit and the only FDA-approved generic SFG product. The BD study was designed to address whether acceptable variations between the innovator and generic products would result in either a difference in BD or a difference in potential for oxidative stress as measured by serum NTBI levels. To associate any measured BD or safety differences to a product characteristic difference, thorough analyses of product physiochemical properties was necessary. The results of these analyses are reported in a separate publication that describes, in detail, the minor differences detected between the products and interprets those differences in the context of a generic product review [12,13]. A pharmacokinetic study [14] was conducted to determine early time points and a dose concentration for the BD study. The BD study organs included blood serum to measure maximal drug iron exposure and its rate of systemic delivery, bone marrow as the designated pharmacological target tissue, lungs, brain, and heart as critical organs of toxicity concern, and spleen, liver, and kidney as the primary organs of uptake, storage, and excretion. 

## 2. Methods

### 2.1. SFG Products

SFG products were obtained from a commercial pharmacy in their retail packaging. Both the innovator (Ferrlecit^®^, Sanofi-Aventis U.S., Inc., Bridgewater, NJ, USA, lot A5075) and the generic (SFG Complex in Sucrose Injection, Watson Pharma Inc., Parsippany, NJ, USA, lot 142290.1) come in 62.5 mg, 5 mL single use vials at a concentration of 12.5 mg/mL. Detailed physicochemical characterizations of the Ferrlecit and generic SFG lots were reported by Sun et al. [12]. Single lots of each product were used for the BD study.

### 2.2. Animal Care and Use

All animal experiments were conducted under an approved protocol and oversight of the Food and Drug Administration’s White Oak Federal Research Center Institutional Animal Care and Use Committee. Animal research was conducted in an AAALAC accredited facility in accordance with the Guide for the Care and Use of Laboratory Animals, 8th Edition [15]. Male Sprague Dawley rats approximately 300 to 350 g were purchased from Taconic Farms (Derwood, MD, USA) with cannulation of one jugular vein. Animals were kept on a 12 h light cycle and received food and water ad libitum. All rats were acclimated for a minimum of 7 days prior to initiation of experiments. 

### 2.3. Experimental Design for Biodistribution Study

Prior to design of the biodistribution study, a pharmacokinetic (PK) study [14] was conducted with the innovator product that demonstrated a returned to baseline by 24 h after dosing. Based on that PK study and the recognized tissue retention of iron from intravenous iron oxide nanoparticle products [16,17], immediate time points (<24 h) were included to assure that similar dosing and PK parameters were obtained, that free iron of potential toxicity was not different and that immediate organ distribution was the same between the innovator and generic. The extended time points (weeks) were to demonstrate no long-term differences between the two products in iron distribution or utilization. Given no reported sex-related discrepancy in PK or in clinical efficacy for the long-used innovator product, only male rats were employed thereby allowing reduction in animal use.

Three equal cohorts were created from a group of 126 male Sprague Dawley rats to form 3 treatment groups of 42 rats with each group receiving a different single treatment via indwelling intravenous cannula. The three treatment groups were: (1) the innovator SFG product dosed intravenously at a concentration of 40 mg/kg; (2) the generic SFG product dosed intravenously at a concentration of 40 mg/kg; (3) saline dosed intravenously at equivalent volume to SFG products given at 40 mg/kg concentration. Following treatment, 6 rats from each group were sacrificed at 15 min, 1 h, 8 h, 1 week, 2 weeks, 4 weeks, and 6 weeks post-treatment.

### 2.4. Animal Necropsy and Tissue Sampling

Each animal was anesthetized via isoflurane induction and maintenance. The abdomen and thorax were opened and the animal sacrificed through exsanguination via the caudal vena cava in conjunction with bilateral pneumothorax. Blood was collected in serum collection tubes and subsequently centrifuged for 20 min at 2000 rpms for serum collection via decantation. This procedure was repeated to remove any remaining cells or debris. The entire brain, kidneys, spleen, heart, liver, and lungs were harvested along with bone marrow from both femurs. All sampled tissues were weighed and prepared for bioanalysis to determine tissue total iron levels. Complete sample sets were obtained from each rat in all treatment groups. A total of 1008 test samples were generated encompassing seven time points (15 min, 1 h, 8 h, 1 week, 2 weeks, 4 weeks, and 6 weeks) from the three treatment cohorts (innovator, generic, control). The measurements of total tissue weights were conducted at the time of collection. Tissue samples were then stored at −80 °C. The day before digestion, samples were moved to −5 °C and thawed before further processing.

### 2.5. Sample Processing by Microwave Digestion

To determine total iron concentration in tissue, smaller tissue samples, approximately 200–400 mg, were collected and weighted from each of the pre-weighed and prepared tissues. The tissue samples for brain, lung, heart, spleen, kidney, and liver were placed in a Teflon vessel and 8 mL of 70% nitric acid (HNO_3_) was added to the vessel. Because of the limited sample size of serum and bone marrow, the digestion volumes were reduced to ensure a detectable level of iron would remain. For serum, 1 mL was pipetted into the Teflon vessel and digested with 4 mL of 70% HNO_3_. For the even more limited bone morrow samples, the entire sample (between 0.028 mg and 0.125 mg) was digested with 3 mL of 70% HNO_3_. All vessels were then capped and sealed using a manual torque cog to allow for off gassing during the high temperature microwave digestion process and to avoid pressure build up in the vessels. The microwave digestion was done using a MARS 6 microwave system (CEM Corporation, Matthews, NC, USA). The samples were microwaved for a total of 30 min, with a 15-min ramp time, slowly heating the samples to 200 °C with interval microwaves at a power of 800 Watts. Once the 200 °C temperature was reached, the samples were held at 200 °C for 15 additional minutes. Following this microwave method, the samples were allowed to cool for an hour before being transferred to a stock sample bottle, and then further diluted to 2% (*v*/*v*) in HNO_3_ and stored in a 15-mL sample tube awaiting ICP-MS analysis.

### 2.6. Iron Quantification via Inductively Coupled-Mass Spectrometry (ICP-MS)

A Perkin Elmer (Shelton, CT, USA) ICP-MS instrument (NexIon 300D) was used to determine the amount of total iron content in the samples. The ICP-MS was interfaced with PrepFast (ESI, Lincoln, NE, USA)—an advanced, automated dilution syringe apparatus. Standard, sample, and quality control (QC) solutions were delivered to the nebulizer via a peristaltic pump at 0.1 mL per minute where the nebulizer converted the sample solution to a spray mist using argon (Ar) gas. Indium (In) was chosen as internal standard and was added during the analysis with the aid of PrepFast by in-line mixing. The PrepFast system was interphased with the ICP-MS and used to auto-dilute the stock solutions to generate the calibration curves and the system QC samples, additional sample QCs were also generated by hand to ensure auto-dilution stability. Three identical stock solutions Ia, Ib and Ic (1000 parts per billion (ppb) of Iron (Fe)) were used to generate three calibration curves by dilution using PrepFast. The dilution factors 200, 100, 20, 10, 5 and 2 generated the corresponding calibration points of 5 ppb, 10 ppb, 50 ppb, 100 ppb, 200 ppb and 500 ppb, respectively. Similarly, four levels of QCs were diluted from stock solution II (1000 ppb Fe) using dilution factors of 200, 100, 10 and 2 to generate 5 ppb (Lower Limit of Quantitation or LLOQ), 10 ppb (Low QC or LQC), 100 ppb (Mid QC or MQC) and 500 ppb (High QC or HQC), respectively. Each level of QC was diluted 5 times to generate 5 QCs at each level.

Two different standard solutions of Fe (1000 ppm) were purchased from two separate sources Perkin Elmer (Shelton, CT, USA) and High Purity Standards (Charleston, SC, USA). Optima grade concentrated HNO_3_ was purchased from Fischer Scientific (Fairlawn, NJ, USA). A solution of 1000 ppm In was purchased from Perkin Elmer (Shelton, CT, USA). Filtered 18 MOhm water was supplied in house by a Millipore Milli-Q System (Bedford, MA, USA). The setup solution to optimize ICP-MS was purchased from Perkin Elmer (Shelton, CT, USA). Preparation of calibration standards and quality control standards are described below.

#### ICP-MS Methods

- *Preparation of Calibration Standards:* Three stock solutions Ia, Ib and Ic (1 ppm or 1000 ppb Fe) were prepared by pipetting 50 µL of 1000 ppm Fe reference standard solution (Perkin Elmer) to a 50-mL volumetric flask and diluting with 2% (*v*/*v*) HNO_3_.
- *Preparation of Quality Control Standards:* The QC Fe stock solution II (1 ppm or 1000 ppb) was prepared by pipetting 100 µL of 1000 ppm metal reference standard solution (High Purity Standards) in a 100 mL volumetric flask and diluting with 2% (*v*/*v*) HNO_3_.
- *Preparation of System Suitability:* System suitability standard was chosen to be in the middle of the calibration range. A stock solution (1 ppm or 1000 ppb Fe) was prepared by pipetting 50 µL of 1000 ppm metal reference standard solution (Perkin Elmer) to a 50-mL volumetric flask and diluting with 2% (*v*/*v*) HNO_3_.
- *Preparation of Dilution System Suitability*: Dilution system suitability quality controls (QC) were chosen to be in the middle of the calibration range. A stock solution (1 ppm or 1000 ppb Fe) was prepared by pipetting 50 µL of 1000 ppm metal reference standard solution (Perkin Elmer) to a 50-mL volumetric flask and diluting with 2% (*v*/*v*) HNO_3_. QC standards were made at 1000 ppb Fe, 500 ppb Fe and 250 ppb Fe by serial dilution and places throughout sample analysis to observe the two fold dilution integrity of the auto-sampler.
- *Preparation of Internal Standard:* Stock solution of 30 ppb In was prepared by pipetting 30 µL of 1000 ppm In metal reference standard solution (Perkin Elmer) to a 2.0 L volumetric flask and diluting with 2% (*v*/*v*) HNO_3_.

### 2.7. ICP-MS Analytical Method Validation

An analytical method for elemental Fe determination was developed and then validated according to compendial methods <1225> and USP <233> [18,19]. The following validation parameters were addressed: linearity, range, accuracy, and precision. The optimized operating conditions used for ICP-MS are summarized in Supplemental Table S1. Validation data for these parameters are presented in Supplemental Tables S2–S5. Validation results for the ICP-MS method for system suitability (*n* = 6) fall within the acceptable limits (Supplemental Table S2) based on guidance requirements.

#### 2.7.1. Linearity and Range

The standard calibration curve was linear over the analytical range of 5–500 ppb of Fe. For validation sets on three days, the results are summarized in Supplemental Table S3 and an acceptable correlation was observed over the analytical range (r^2^ ≥ 0.9996).

#### 2.7.2. Accuracy and Precision

Accuracy is the closeness of mean test results obtained by the method to the true value of the analyte calculated as percent recovery. Precision describes the degree of agreement among individual test results when the method is applied repeatedly to multiple samplings of a homogenous sample and is calculated as relative standard deviation (R.S.D.). The results of intra-day accuracy and precision in the study are compiled in Supplemental Tables S4 and S5. Both accuracy and precision meet FDA acceptance criteria [20].

### 2.8. Biodistribution Characterization

All biodistribution samples were analyzed using the PrepFast autosampler and its advanced, automated dilution syringe apparatus interfaced with the ICP-MS. Endogenous iron levels were measured in control rats at each biodistribution time point to monitor iron concentration changes with age and diet. After ICP-MS analysis, the resulting biodistribution sample concentrations were characterized as group mean tissue iron concentration per tissue weight and as group mean total iron (whole tissue) content for each tissue of all groups at every time point. The standard deviation (SD) and coefficient of variation (CV) were calculated and the control group’s mean iron concentrations were subtracted from the innovator and generic group concentrations as background or baseline allowing direct comparison of drug induced iron concentration between the drug products. 

### 2.9. Statistical Analysis

Mean tissue iron concentrations and standard deviations were calculated for each treatment (innovator, generic, control) at each time point. Control means were then subtracted from innovator and generic means to isolate drug related iron concentrations. Standard deviations for innovator or generic means were added to the standard deviation of the control mean to acquire a standard deviation for the difference between the means. These adjusted means and standard deviations were used to determine if there were differences between treatments across the entire experimental time line using comparison of means calculator (https://www.medcalc.org/calc/comparison_of_means.php) [21,22]. A statistical comparison of proportions was used to test the hypothesis that there is no difference between group means of innovator and generic area-under-the-curve (AUC) for each tissue (https://www.medcalc.org/calc/comparison_of_proportions.php) [19]. 

## 3. Results

**Figure 1:**
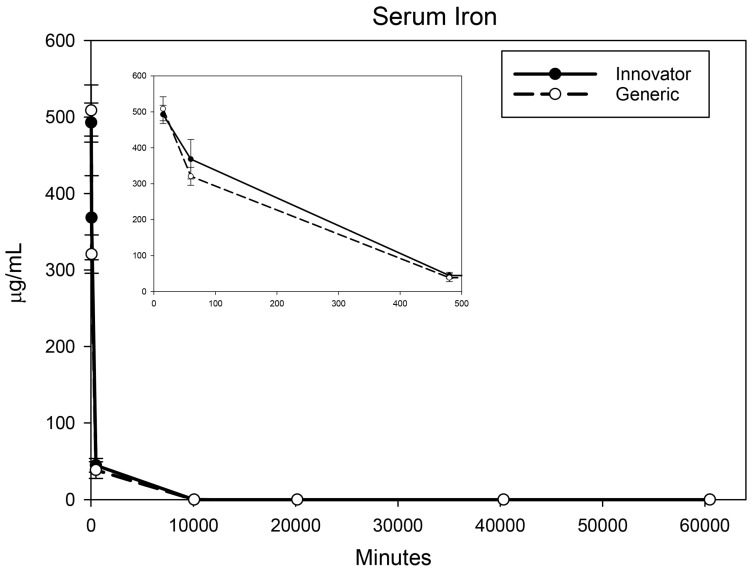
Serum mean iron concentrations over time. Endogenous (control) iron concentration was subtracted from both the innovator (solid) and the generic (dashed). Negative results are not graphed. Time points = 15 min, 60 min (1 h), 480 min (8 h), 10,080 min (1 week), 20,160 min (2 weeks), 40,320 min (4 weeks), 60,480 min (6 weeks). Inset shows the first three time points (15 min, 1 h, 8 h). Values are shown as mean + SD; *n* = 6 per group per time point.

**Figure 2:**
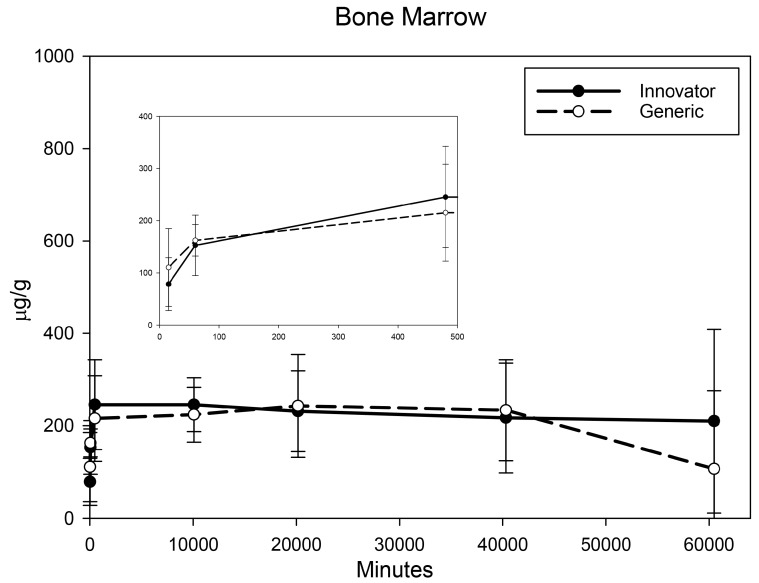
Bone marrow mean iron concentrations over time. Endogenous (control) iron concentration was subtracted from both the innovator (solid) and the generic (dashed). Negative results are not graphed. Time points = 15 min, 60 min (1 h), 480 min (8 h), 10,080 min (1 week), 20,160 min (2 weeks), 40,320 min (4 weeks), 60,480 min (6 weeks). Inset shows the first three time points (15 min, 1 h, 8 h). Values are shown as mean + SD; *n* = 6 per group per time point.

**Figure 3:**
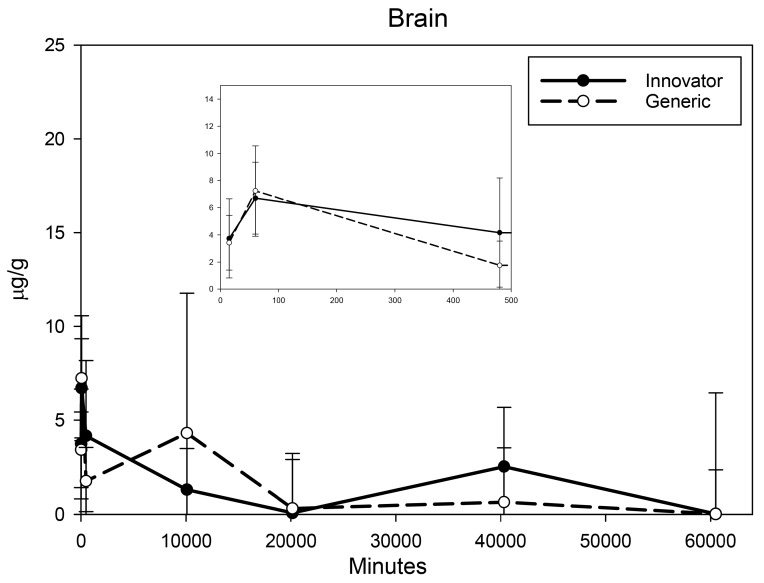
Brain mean iron concentrations over time. Endogenous (control) iron concentration was subtracted from both the innovator (solid) and the generic (dashed). Negative results are not graphed. Time points = 15 min, 60 min (1 h), 480 min (8 h), 10,080 min (1 week), 20,160 min (2 weeks), 40,320 min (4 weeks), 60,480 min (6 weeks). Inset shows the first three time points (15 min, 1 h, 8 h). Values are shown as mean + SD; *n* = 6 per group per time point.

**Figure 4:**
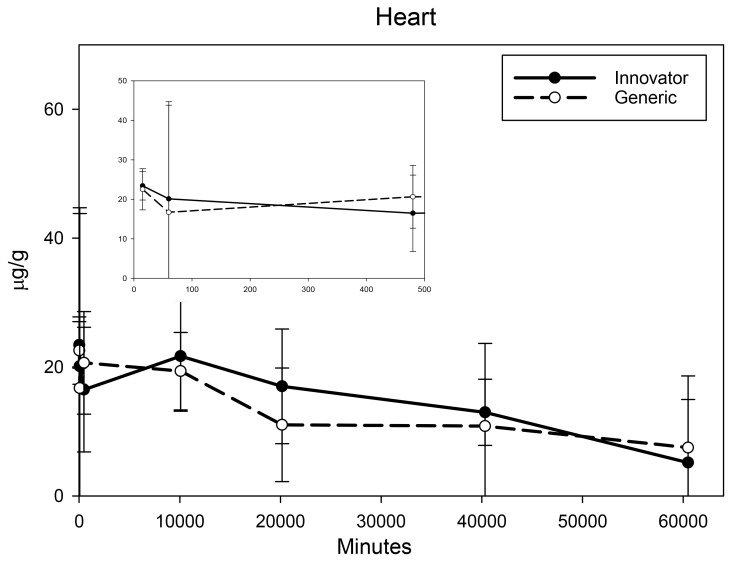
Heart mean iron concentrations over time. Endogenous (control) iron concentration was subtracted from both the innovator (solid) and the generic (dashed). Negative results are not graphed. Time points = 15 min, 60 min (1 h), 480 min (8 h), 10,080 min (1 week), 20,160 min (2 weeks), 40,320 min (4 weeks), 60,480 min (6 weeks). Inset shows the first three time points (15 min, 1 h, 8 h). Values are shown as mean + SD; *n* = 6 per group per time point.

**Figure 5:**
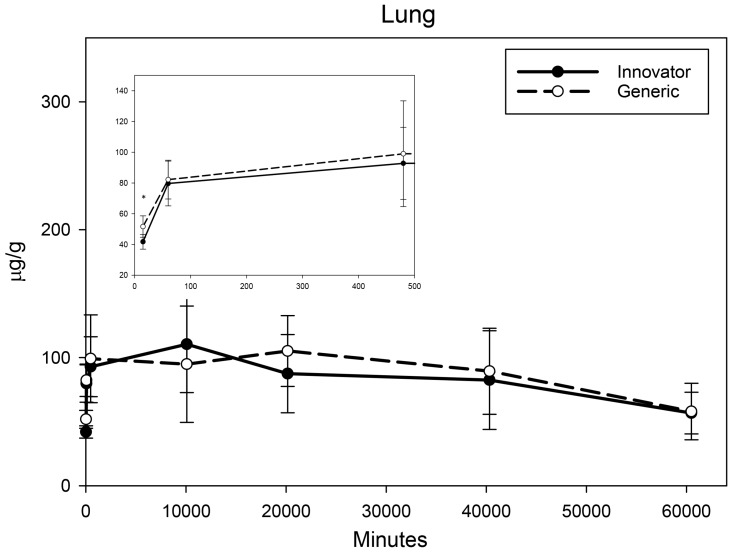
Lung mean iron concentrations over time. Endogenous (control) iron concentration was subtracted from both the innovator (solid) and the generic (dashed). Negative results are not graphed. Time points = 15 min, 60 min (1 h), 480 min (8 h), 10,080 min (1 week), 20160 min (2 weeks), 40,320 min (4 weeks), 60,480 min (6 weeks). Inset shows the first three time points (15 min, 1 h, 8 h). Values are shown as mean + SD; *n* = 6 per group per time point; * *p* < 0.05.

**Figure 6:**
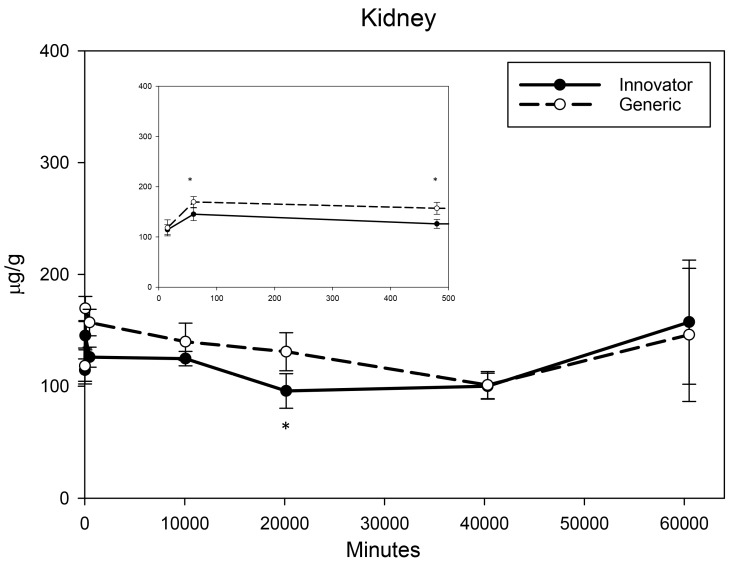
Kidney mean iron concentrations over time. Endogenous (control) iron concentration was subtracted from both the innovator (solid) and the generic (dashed). Negative results are not graphed. Time points = 15 min, 60 min (1 h), 480 min (8 h), 10,080 min (1 week), 20,160 min (2 weeks), 40,320 min (4 weeks), 60,480 min (6 weeks). Inset shows the first three time points (15 min, 1 h, 8 h). Values are shown as mean + SD; *n* = 6 per group per time point; * *p* < 0.05.

**Figure 7:**
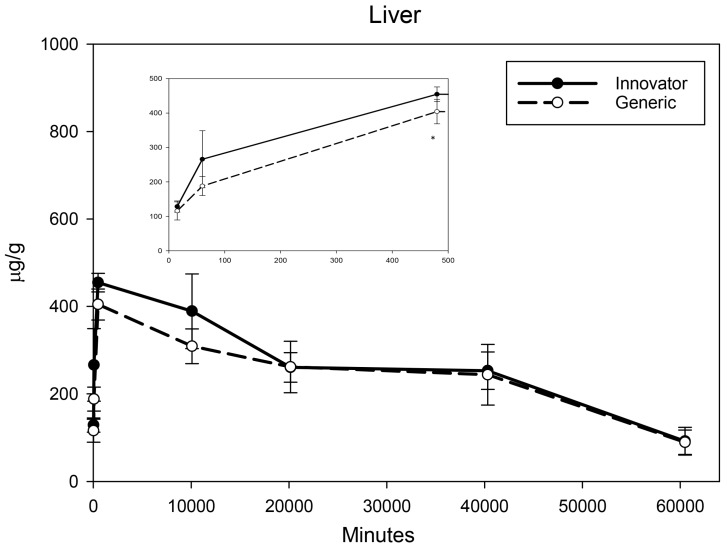
Liver mean iron concentrations over time. Endogenous (control) iron concentration was subtracted from both the innovator (solid) and the generic (dashed). Negative results are not graphed. Time points = 15 min, 60 min (1 h), 480 min (8 h), 10,080 min (1 week), 20,160 min (2 weeks), 40,320 min (4 weeks), 60,480 min (6 weeks). Inset shows the first three time points (15 min, 1 h, 8 h). Values are shown as mean + SD; *n* = 6 per group per time point; * *p* < 0.05.

**Figure 8:**
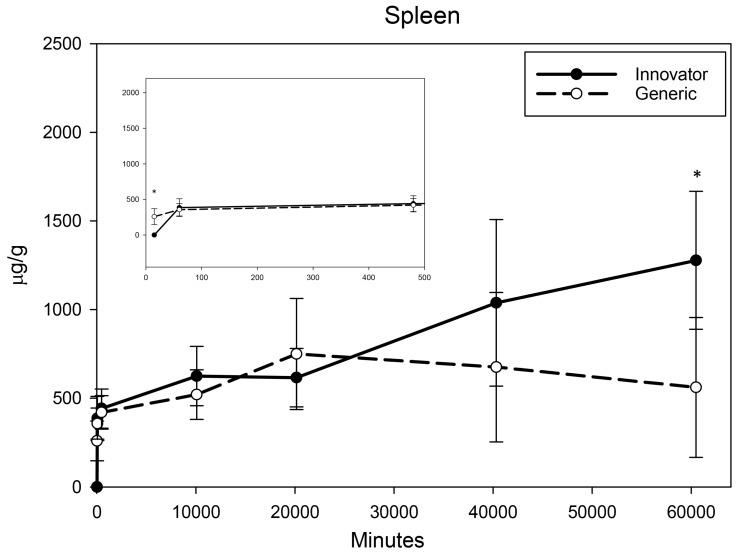
Spleen mean iron concentrations over time. Endogenous (control) iron concentration was subtracted from both the innovator (solid) and the generic (dashed). Negative results are not graphed. Time points = 15 min, 60 min (1 h), 480 min (8 h), 10,080 min (1 week), 20,160 min (2 weeks), 40,320 min (4 weeks), 60,480 min (6 weeks). Inset shows the first three time points (15 min, 1 h, 8 h). Values are shown as mean + SD; *n* = 6 per group per time point; * *p* < 0.05.

All biodistribution samples analyzed by ICP-MS are expressed as mean tissue iron concentrations (µg/g) and mean total iron (whole tissue) content (µg/tissue) for each tissue of all groups at each study time point are shown in Supplemental Worksheet 1. Measurable iron concentrations were found in all samples from all time points. The standard deviation (SD) and coefficient of variation (CV) were calculated and for each time point and the control group’s mean iron concentration was subtracted from the innovator and generic group mean concentrations with the resultant values graphically represented in individual organ comparisons (Figure 1, Figure 2, Figure 3, Figure 4, Figure 5, Figure 6, Figure 7 and Figure 8). Innovator and generic SFG product tissue iron concentration data were also plotted as a percent of total detected iron. These values are graphically presented in Figure 4.

### 3.1. Pharmacological Delivery and Target Tissue

The iron concentration in serum was measured by ICP-MS. Concentrations including any remaining drug iron for both products across the first three time points coincided with measurements from the previous pharmacokinetic study [13]. As expected, iron concentration in the serum was greatly elevated for both products at the first post-treatment time point (15 min) followed by almost complete clearance from blood by 8 h post-treatment (Figure 1). No significant differences were observed at any time point. The targeted tissue for pharmacological effect for sodium ferric gluconate drug is the bone marrow. The iron concentration there quickly increased and peaked by 8 h after dosing and then remained stable over the remaining six weeks of the study. No significant concentration differences between the two products were determined in the bone marrow (Figure 2).

### 3.2. Sensitive Tissues of Toxicity Concern

Brain, heart, and lungs were analyzed as tissues that might be more susceptible to toxicity induced by acute oxidative stress due to excess iron intake. Drug induced increases in iron concentration of these organs were relatively small. In the brain, an extremely small increase in iron concentration above control was seen with both SFG products at 15 min and 1 h. At 8 h and beyond, there was no increase in iron concentration relative to the range of concentrations seen in controls and there was no difference in distribution detected between the two products (Figure 3). Iron concentration in the heart peaked by 15 min and remained elevated throughout the six-week study (Figure 4) with a decreasing trend that did not differ between the two SFG products. The lung iron concentration increased over the first 8 h then remained relatively stable decreasing only slightly over the six-week biodistribution study (Figure 5). A significant difference between the innovator and generic products in iron distribution in the lungs was noted only at 15 min post-treatment.

### 3.3. Uptake, Storage, and Excretion Tissues

Given their role in iron uptake, storage, and excretion, much larger increases in iron concentrations were anticipated in the kidney, liver, and spleen. The kidneys experienced moderate, immediate, and persistent iron concentration elevation (Figure 6). Analyses indicated slightly higher levels of iron at 1 h, 8 h, and 2 weeks in the kidneys of rats treated with the generic formulation (with a non-significant but similar trend at one week). At four and six weeks, no product related differences in iron concentration were detected in the kidneys. Iron concentration in the liver elevated over the first three time points then slowly decreased over the remaining five weeks (Figure 7) with both products. Statistical interrogation revealed slightly higher iron concentrations in the livers of rats given the innovator compared to the generic only at 8 h post-treatment. At subsequent time points, no differences in iron distribution between the products were detected in liver iron concentrations. For both products, iron concentration in the spleen was shown to increase over the first two weeks (Figure 8). At four and six weeks, the spleen iron concentration in the generic SFG treated group remained level or decreased slightly while the iron concentration of the innovator SFG treated group continued to increase resulting in statistically higher iron concentrations in the spleens of the innovator group at six-weeks post-treatment. A steady but slow increase of iron concentration was also observed in the spleens of the control group (data not shown). These escalating control values were consistently subtracted from innovator and generic values. Over these three organs, there were statistically significant differences in iron concentration between the innovator and generic products at a very limited number of isolated time points. 

### 3.4. Total Percent Distribution of Iron

**Figure 9:**
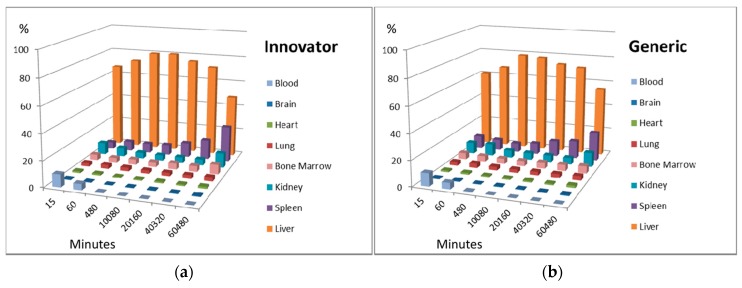
Percent distribution of the innovator and genetic drug iron concentration among the analyzed organs; (**a**) innovator drug; (**b**) generic drug; *n* = 6 per group per time point.

To better appreciate the overall iron distribution of these complex SFG drug products, this data was presented as a percent of total measured iron (iron measured by ICP-MS across all examined tissues). This was calculated by multiplying the iron concentration in a tissue by the measured mass of that tissue to get total tissue iron content. By adding all of these tissue contents, a total measured iron value was derived. Dividing the total iron content for each tissue by the total measured iron value allowed calculation of the percent of iron distributed to each tissue. The percentages of iron distribution of the innovator and generic SFG products to the assessed tissues were plotted and placed side by side in Figure 9 to better display and compare overall distribution. This approach does not provide for statistical comparison but visual inspection suggests no large differences in overall distribution based on SFG product treatment.

### 3.5. Total Area-Under-the-Curve (AUC)

**Figure 10:**
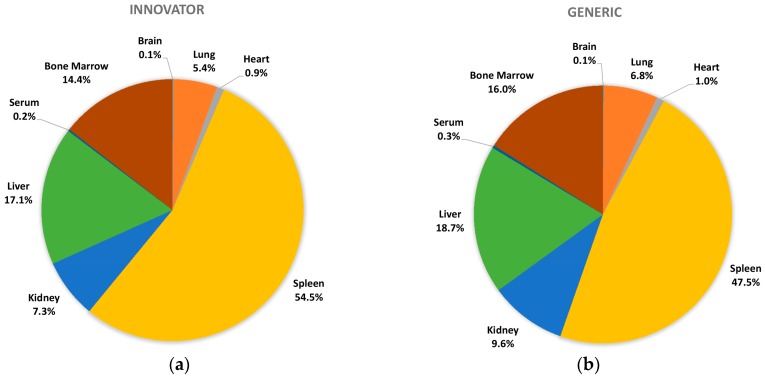
AUC calculated using Phoenix WinNonlin; for tissues: AUC calculated from 0 to 60,480 min—where conc. at t_0_ = 0 µg/g; for serum after subtraction control values from both (**a**) innovator and (**b**) generic drug values.

Another approach to interpreting similarity or difference in iron exposure in each of the rat organs is evaluation of the total area-under-the-curve (AUC_0–60,480 min_) for iron concentrations of each product in each tissue across the entire time course of the study. Figure 10 provides a graphic comparison of the AUC values calculated from the distribution concentrations measured in each tissue in this study. The spleen appeared to be the only tissue with considerable difference (7%) in iron concentrations based on exposure to different SFG products, however even this difference was not statistically significant.

## 4. Discussion

Spurred by discrepancies between FDA’s 2013 Draft Guidance on Sodium Ferric Gluconate Complex [9] and the EMA’s 2011 Draft Reflection Paper on the Data Requirements for Intravenous Iron-based Nano-colloidal Products Developed with Reference to an Innovator Medicinal Product [11], the two FDA approved SFG products were investigated in a comprehensive BD study, confirming no significant and biologically relevant differences. Prior to initiating this BD study, product physiochemical characterization [12], cellular uptake [13], and pharmacokinetic [14] studies were completed to inform the BD study design. The BD study incorporated serum and bone marrow to monitor pharmacological delivery and target tissues. In addition, highly sensitive organs (brain, heart, lungs) and bio-accumulation/excretion organs (kidney, liver, spleen) were monitored for iron concentration levels. Although there were existing iron detection methods for plasma and serum, those methods were not easily adapted for tissue analysis [23,24,25,26,27]. Thus, a tissue digestion and analytical ICP-MS detection method were developed to accurately measure iron concentration in tissue samples. This method was universally used for all tissues with small adjustment to the serum and bone marrow digestion due to small sample volumes. Since ICP-MS detection is indiscriminate to iron species and source, endogenous iron would be present in every sample. To address the endogenous iron concentration, a control group was used to obtain iron baseline levels. All biodistribution data were acquired by ICP-MS analysis of digested tissue samples. The ICP-MS analytical method was validated according to compendial methods <1225> and USP <233> based on FDA guidance [18,19,20]. 

This biodistribution study directly compared iron concentrations in major organ tissues of rats parenterally administered with the innovator and generic SFG drug complexes. All measurements were obtained with a validated bioanalytical method and were similar in magnitude and variability to studies with related products [19]. The overall tissue distribution of the two drugs was very similar. The early time point kinetics of both SFG drugs in serum were similar to those observed previously in the pharmacokinetic study [14]. At some time points, differences in product related iron concentration was observed in one or two tissues. However, no difference in overall distribution was observed nor any consistent difference in distribution to any tissue between two SFG products. At any given time point, the overall percent iron concentration distribution did not vary by more than 7% in any organ between two SFG products. The individual tissues that exhibited statistically different iron concentrations between the innovator and generic SFG products at specific time points generally lost these differences at subsequent time points as concentrations converged. Trends suggested that kidney uptake and/or excretion may have initially been slightly larger with the generic product and liver uptake and/or excretion may have initially been slightly larger with the innovator. At 4 and 6 weeks, neither of these initial trends was evident. Further, the small magnitude of these trends does not support biological relevance and makes it unclear as to whether these trends would be reproducible. 

At the six-week time point, mean comparisons indicate differences between the innovator and generic SFG product in accumulation of iron in the spleen. However, total organ iron content, percent of total iron distribution, and AUC analyses across the entire timeline did not reveal an overall disparity in distribution to the spleen. Closer investigation revealed that rats receiving generics had consistently larger spleens than the control group while the rats receiving innovator product had spleens that were slightly but consistently smaller than rats in the control group. The reason for and the relevance of this unanticipated finding were not identified. A steady but slow increase of iron concentration was observed in the spleens of the control group as well but this was expected based on one literature results on a study of normal physiological changes in rats due to aging [28]. 

The presence of these small differences only in the excretion organs suggests a slight difference between the products in how iron might be excreted and/or stored as opposed to differences in tissue distribution. Based on this study’s findings including the lack of differences in iron distribution to the bone marrow and tissues of acute toxicity concern (brain, heart, lung) and the small magnitude of the observed differences in the kidney, liver, and spleen, there is no evidence of difference in biodistribution between the products sufficient to impact the safety profiles. 

## 5. Conclusions

This biodistribution study in Sprague Dawley rats compared two FDA-approved SFG colloidal iron products and found that overall tissue distribution was very similar between the innovator and generic products. This biodistribution study revealed no additional potential for differences in toxicity. The results of this biodistribution study further confirms that the current FDA’s bioequivalence approach for SFG complex drug products (i.e., Q1/Q2 formulation sameness, in vivo pharmacokinetic bioequivalence studies, in vitro physicochemical characterization) is sufficiently sensitive to capture potential differences between brand and generic formulations in the absence of any non-clinical biodistribution study.

## Supplementary Materials

The following are available online at www.mdpi.com/2079-1991/8/1/10/s1, Table S1: ICP-MS Parameters, Table S2: Parameters of system suitability, Table S3: Parameters of calibration curve, Table S4: Quality Control: Accuracy (%), Table S5: Quality Control: Precision (% R.S.D.), Worksheet S1: Biodistribution.
