# Supplementary material for: Comparative Evaluation of U.S. Brand and Generic Intravenous Sodium Ferric Gluconate Complex in Sucrose Injection: Biodistribution after Intravenous Dosing in Rats
Source: Nanomaterials (Basel). 2017 Dec 28;8(1):10. doi: 10.3390/nano8010010 (PMC5791097; doi:10.3390/nano8010010)
Supplement: Supplementary file 1 [file nanomaterials-08-00010-s001.zip › nanomaterials-252409-supplementary.pdf]

Table S1: ICP-MS Parameters

ICP Parameters	
RF Power	1600 W
Plasma gas flow rate	18.0 L/min.
Auxiliary gas flow rate	1.2 L/min.
Nebulizer gas flow rate	1.0 L/min.
Spray chamber	Peltier-cooled (2 °C) baffled quartz cyclonic spray chamber
Torch	Quartz
Sampler and skimmer cones	Platinum
Hyper skimmer cones	Nickel
Mass Spectrometer Parameters	
Resolution	0.7 amu at 10% peak maximum
Dwell time	50 ms
Sweeps	20
Readings	1
Replicates	3
Autolens	ON
Internal Standard	In (115 amu)
Mode of detection	KED

Table S2: Parameters of system suitability

		Day 1		Day 2		Day 3	
**Parameter**	**Acceptance Criteria (% RSD)**	**Mean**	**% RSD**	**Mean**	**% RSD**	**Mean**	**% RSD**
**Fe (cps)**	NMT 10 %	82341.5	0.36	74296.8	0.42	69909.6	0.71
**In (Internal Standard, cps)**	NMT 10 %	16914.8	0.34	15309.6	0.48	14601.8	0.70
**Ratio (Fe/In)**	NMT 2 %	4.8	0.09	4.8	0.25	4.7	0.42

Table S3: Parameters of calibration curve

**Calibration sets**	**Linear Range (Fe ppb)**	**Calibrators**	**Correlation coefficient (r^2)**	**Slope**
1	5-500	6	0.9999	0.0246
2	5-500	6	0.9999	0.0244
3	5-500	6	0.9999	0.0242

Table S4: Quality Control: Accuracy (%)

S. No.	5 ppb	50 ppb	200 ppb	500 ppb
Day 1	93.5	96.4	98.1	97.8
Day 2	91.9	96.8	98.5	98.9
Day 3	106.6	98.6	98.4	97.0

Table S5: Quality Control: Precision (% R.S.D.)

S. No.	5 ppb	50 ppb	200 ppb	500 ppb
Day 1	4.9	0.6	0.1	0.4
Day 2	4.76	0.58	0.25	1.25
Day 3	1.03	0.31	0.42	0.95
